# Crop Protection against *Botrytis cinerea* by Rhizhosphere Biological Control Agent *Bacillus velezensis* XT1

**DOI:** 10.3390/microorganisms8070992

**Published:** 2020-07-03

**Authors:** Laura Toral, Miguel Rodríguez, Victoria Béjar, Inmaculada Sampedro

**Affiliations:** 1 Xtrem Biotech S.L., European Business Innovation Center, Avenida de la Innovación, 1, Armilla, 18016 Granada, Spain; 2 Department of Microbiology, Faculty of Pharmacy, Campus de Cartuja s/n, 18071 Granada, Spain; miguelrg@correo.ugr.es (M.R.); vbejar@ugr.es (V.B.); 3 Biomedical Research Center (CIBM), Institute of Biotechnology, Avenida del Conocimiento s/n, Armilla, 18100 Granada, Spain

**Keywords:** biocontrol, *Botrytis*, *Bacillus*, PGPR, phytohormones, callose, H_2_O_2_, MDA, tomato, strawberry

## Abstract

This study aims to evaluate the use of *Bacillus velezensis* strain XT1 as a plant growth-promoting rhizobacterium (PGPR) and biocontrol agent against *B. cinerea* in tomato and strawberry plants. Foliar and radicular applications of strain XT1 increased plant total biomass as compared to the control and *B. cinerea*-infected plants, with root applications being, on the whole, the most effective mode of treatment. Applications of the bacterium were found to reduce infection parameters such as disease incidence and severity by 50% and 60%, respectively. We analyzed stress parameters and phytohormone content in order to evaluate the capacity of XT1 to activate the defense system through phytohormonal regulation. Overall, the application of XT1 reduced oxidative damage, while the H_2_O_2_ and malondialdehyde (MDA) content was lower in XT1-treated and *B. cinerea*-infected plants as compared to non-XT1-treated plants. Moreover, treatment with XT1 induced callose deposition, thus boosting the response to pathogenic infection. The results of this study suggest that the signaling and activation pathways involved in defense mechanisms are mediated by jasmonic acid (JA) and ethylene hormones, which are induced by preventive treatment with XT1. The study also highlights the potential of preventive applications of strain XT1 to activate defense mechanisms in strawberry and tomato plants through hormone regulation.

## 1. Introduction

Increasing human populations and demand for food to meet basic requirements are a major challenge for agriculture. In addition to the difficulty of meeting these demands, there is the damage to crops caused by pathogens [1]. Pathogen infections have been estimated to cause a 10–16% loss in food crops worldwide, which, in economic terms, amounts to 200 million euros annually [2].

Pathogens that cause major economic losses include the necrotrophic fungus *Botrytis cinerea.* This fungus, which is known to cause gray mold, affects a wide range of host plants and infects over 200 plant species, including important horticultural crops [3,4]. The disease process of *B. cinerea* is affected by many pathogenicity determinants, such as host cell wall degradation, cell death and host defense avoidance [5,6,7,8,9,10]. One of the most important determinants is the production of an exopolysaccharide (EPS) of the β-(1,3)(1,6)-D-glucan type, which induces salicylic acid (SA) production and antagonizes optimal signaling pathways for plant defenses against *B. cinerea* and the synthesis of enzymes, such as copper–zinc superoxide dismutase, that contribute to H_2_O_2_ generation [8,9,10]. Moreover, its ability to produce conidia enables *B. cinerea* to remain quiescent until conditions are sufficiently favorable to produce the infection [3]. Low host specificity also enables *B. cinerea* to affect a large number of crops worldwide, including tomato and strawberry, both of which are of great economic importance in Spain, where the largest economic losses caused by *B. cinerea* occur during the harvest and storage season [11]. According to the Spanish Ministry of Agriculture, Fisheries and Food (MAPA), in the 2017/2018 period, Spain was one of the largest producers of strawberries in Europe (26%), with exports of 461.3 million euros, and of tomatoes in the world, with 4.7 million hectares devoted to this crop. In 2016, world tomato production reached 177 million tons according to the Food and Agriculture Organization of the United Nations Statistics Division (FAOAST), with Spain accounting for around 812,571 tons (MAPA) [12,13].

Currently, agricultural management practices are mainly based on the use of chemical pesticides and fertilizers. However, the excessive use of these products has led to the accumulation of residues that are harmful to the environment and humans [14], which has led to the emergence of microbial control agents as an alternative to chemical approaches. These microorganisms, also referred to as plant growth-promoting rhizobacteria (PGPR), which inhibit pathogenic infections, sometimes increase plant growth [15,16,17]. Strains belonging to the genus *Bacillus,* such as *Bacillus subtilis*, *B. thuringiensis*, *B. amyloliquefaciens* and *B. megaterium*, employed in the formulation of PGPR products, are currently used by the agricultural industry to combat and manage pests [18]. In recent decades, the species *B. velezensis* [19] has become one of the most, if not the most, commonly used PGPR [20]. Some of the strains belonging to this species are capable of producing a wide variety of metabolites, such as siderophores [21], enzymes [18], volatile organic compounds (VOC) and lipopeptides [22,23,24,25,26,27,28], which contribute to both their biofertilizing activity and biocontrol capacity through direct competition, by activating induced systemic resistance (ISR) and by modifying the host–plant microbiome [29].

One of the biological control systems developed by plants, which play an important role in protecting against pathogenic infections, is induced systemic resistance (ISR), which activates different signal transduction pathways [30]. A large number of non-pathogenic rhizobacterial species have the ability to trigger ISR by producing elicitors which are perceived by plants and reinforce their defensive systems [31,32,33]. ISR-activated defense responses include the accumulation of callose deposits on cell walls [34,35] and the production of hydrogen peroxide (H_2_O_2_) [36,37]. The phytohormones salicylic acid (SA), jasmonic acid (JA), abscisic acid (ABA) and ethylene (ET) play a fundamental role in the initiation and regulation of these defense mechanisms [38,39,40]. The coordination of defenses against biotrophic pathogens is generally mediated by the action of SA, while the JA/ET tandem is associated with protection against necrotrophic pathogens such as *Botrytis* [41]. The regulation of both SA and JA/ET systems has been reported, in some cases, to have an antagonistic effect, with the activation of SA increasing susceptibility to necrotrophic pathogens [8].

With the aim of marketing a biological product with plant growth promotion (PGP) potential and antifungal activity against *B. cinerea*, this study evaluated the biocontrol activity of *B. velezensis* strain XT1 CECT 8661 against the necrotrophic fungus *B. cinerea* in tomato and strawberry plants. To that end, we analyzed the activation of defense mechanisms in tomato and strawberry plants, as well as the induced resistance mechanisms involved in these responses.

## 2. Materials and Methods 

### 2.1. Bacterial and Fungal Strains, Media and Culture Conditions

The patented *Bacillus velezensis* strain XT1 (CECT 8861^T^) under license to Xtrem Biotech S.L. (ES2639375B1) was used in this study [19]. This strain was isolated from the *Juncus effusus* rhizosphere in a saline habitat located in Málaga (Spain). The fungal pathogen *Botrytis cinerea*, which was isolated from a Syrah vineyard, was kindly provided by the University of Zaragoza (Spain). Bacterial strains were cultured in a nutrient broth (0.1% (*w*/*v*) meat extract, 0.2% (*w*/*v*) yeast extract, 0.5% (*w*/*v*) peptone, 0.5% (*w*/*v*) NaCl) at 28 °C and in a rotary shaker at 120 rpm for 48 h unless stated otherwise. *B. cinerea* was grown in potato dextrose medium (Difco^®^) at 21 °C for 15 days.

### 2.2. In Vivo Evaluation of the Antifungal Activity of XT1 Strain against B. cinerea

In order to determine the efficiency of different modes of treatment, foliar spraying and root irrigation were used to apply strain XT1 to tomato (*Solanum lycopersicum* L. cv. Mina) and strawberry (*Fragaria ananassa* cv. San Andreas) plants. Both types of plants were cultured in 1 L pots filled with sterile potting soil (Compo Sana universal substrate, Compo, Münster, Germany) and placed randomly in a greenhouse at 25 °C/20 °C (day/night), relative humidity 70–90%, a long-day photoperiod (16:8 h light:dark) and a luminosity of 250 μS cm^−2^ s^−1^. Four conditions were assayed for each type of application: sterile distilled water as non-inoculated treatment (C); *B. cinerea*-infected non-treated plants (Bc); XT1-treated plants (XT1); as well as *B. cinerea* + strain XT1 (XT1+Bc). The plants were divided into three separate replicates per treatment, each with 12 plants. Briefly, 7 days after sowing, each plant of the treatments XT1 and XT1-Bc was firstly foliar sprayed or root irrigated, respectively, with 150 mL of a 1/5 and 1/20 diluted five-day-old culture of strain XT1 (10^8^ CFU mL^−1^) in sterile distilled water in PLOM medium (developed by Xtrem Biotech S.L., under industrial secret). Seven days later, Bc and XT1-Bc plants were foliar sprayed and root irrigated with a conidial suspension of *B. cinerea* (10^6^ conidia mL^−1^). Two days later, a 1/5 and 1/40 dilution of a five-day-old culture of strain XT1 was applied, in XT1 and XT1-Bc treatments, using foliar spraying or root irrigation, respectively. The first inoculation was carried out to study the improvement of the resistance of the plant to invasion by the pathogen, and the second as a mechanism of direct antifungal activity against *B. cinerea*.

Seven days later, the plants were harvested and aerial and radicular parts were weighed for each treatment. Infection symptoms were examined and disease incidence (% of plant infected) and severity in terms of chlorosis, withering, necrosis and defoliation was determined using a 0–4 severity scale: 0, healthy plant; 1, damage 1–33%; 2, damage 34–66%; 3, damage 67–99% and 4, dead plant [42]. For each experiment, a total of ten plants were used per treatment.

Physiological parameters were determined for each treatment. A pool of foliar samples belonging to all plants were ground with liquid nitrogen and from the mixture, three technical replicates were performed. Hydrogen peroxide content was measured at 415 nm following the extraction of foliar samples with cold acetone [43]. Membrane lipid peroxidation was measured by malondialdehyde (MDA) quantification [44]. Callose deposition in fresh leaves was analyzed by fluorescence microscopy (380 nm) following ethanol discoloration and methylene blue staining [45]. Finally, the content of the phythormones ethylene, cytokinin, auxin, gibberellin, abcisic acid (ABA), jasmonic acid (JA) and salicylic acid (SA) in plant tissue was determined by ultrahigh-performance liquid chromatography–mass spectrometry (UPLC–MS) according to the protocol described by Stevens and Berry (1988) [46].

### 2.3. Protection by XT1 against Damage Caused by B. cinerea in Tomato and Strawberry Leaves

From each treatment (C, XT1, Bc or XT1+Bc), tomato and strawberry leaves were placed on square petri dishes (120 × 120 mm) containing a layer of sterile glass beads (Ø 4 mm) and 25 mL of sterile distilled water to maintain a humidity of 80%. Three incisions per leaf were made using a scalpel, and each wound was inoculated with 15 μL of *B. cinerea* (10^6^ conidia mL^−1^). The petri dishes were sealed with parafilm and incubated at 21 °C for 3 days. Signs of necrosis (brown spots) and chlorosis were then evaluated. Three technical replicates with two leaves in each one were performed for each treatment.

### 2.4. Statistical Analysis

Variance analysis was performed using ANOVA and the Kruskal–Wallis test (*p* ≤ 0.05). Significant differences between treatments were analyzed with the aid of Fisher’s least significant difference (LSD) test (*p* ≤ 0.05). Significance was expressed as: *, *p* ≤ 0.05; **, *p* ≤ 0.01; ***, *p* ≤ 0.001; NS, non-significant. All analyses were carried out using Statgraphics Centurion XVI (StatPoint Technologies, Inc., Warrenton, VA, USA) and GraphPad Prism 7.04 (GraphPad Software, San Diego, CA, USA) software.

## 3. Results

Tomato and strawberry experiments under greenhouse conditions were carried out to evaluate the effectiveness of the foliar spraying or root irrigation of *B. velezensis* strain XT1 in promoting growth and in suppressing gray mold disease caused by *B. cinerea.*

### 3.1. Growth Parameters

**Figure 1:**
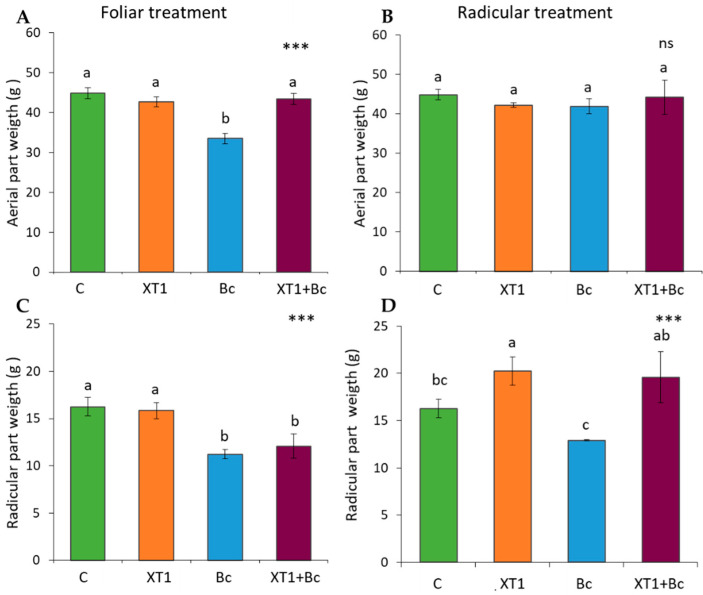
Effect of XT1 foliar (**A**,**C**) and radicular (**B**,**D**) applications on tomato plants. C: non-inoculated control plants; XT1: XT1-treated plants; Bc: *B. cinerea*-infected non-treated plants; XT1+Bc: XT1-treated *B. cinerea*-infected plants. Letters above the main bars represent statistical differences according to the Fisher test *** (*p* ≤ 0.001), ns (no significance). Significant differences between treatments are indicated by different letters (a,b,c).

The plant growth promotion capacity of foliar sprayed and root irrigated strain XT1 was analyzed in tomato and strawberry plants by determining aerial and radicular weight. In tomato plants, we observed plant growth promotion activity following radicular applications of XT1, as indicated by a 20% increase in radicular weight (*p* ≤ 0.001) with respect to the non-inoculated plant (C). This increase was higher (34%, *p* ≤ 0.001) in XT1-treated and *B. cinerea*-infected plants (XT1+Bc) as compared to *B. cinerea*-infected plants (Bc) (Figure 1). Nevertheless, although the appearance of infected plants (Bc) differed considerably from that of XT1-treated infected plants (XT1+Bc), no significant differences in foliar weight were found following treatment. Foliar applications of strain XT1 showed no significant differences in foliar or radicular weight between non-inoculated plants (C) and XT1-treated plants (XT1) (Figure 1).

**Figure 2:**
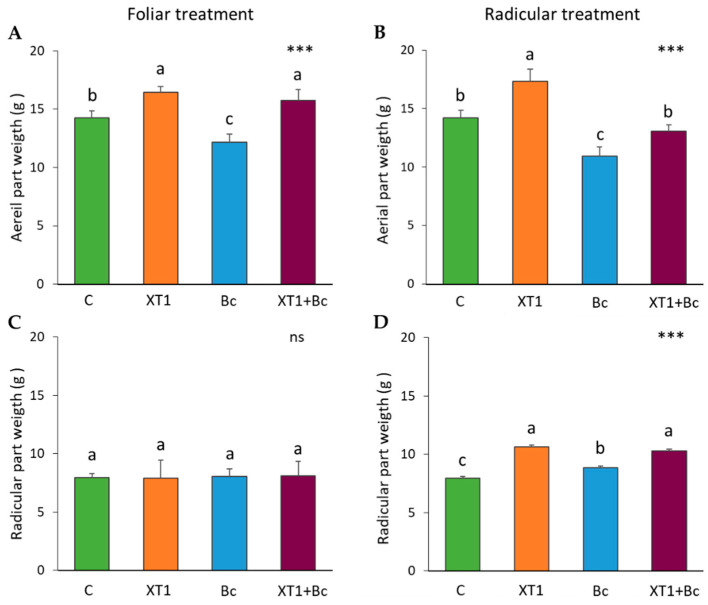
Plant growth promotion (PGP) effect of XT1 foliar (**A**,**C**) and radicular (**B**,**D**) applications on strawberry plants. C: non-inoculated control plants; XT1: XT1-treated plants; Bc: *B. cinerea*-infected non-treated plants; XT1+Bc: XT1-treated *B. cinerea*-infected plants. Letters above the main bars represent statistical differences according to the Fisher test *** (*p* ≤ 0.001), ns (no significance). Significant differences between treatments are indicated by different letters (a,b,c).

Similar results were observed for both non-infected and infected strawberry plants following radicular applications of XT1, with root biomass increasing by 30% with respect to their respective non-inoculated controls (C) and *B. cinerea*-infected non-treated plants (Bc) (Figure 2D). On the other hand, we also observed plant growth promotion activity following radicular (Figure 2B) and foliar applications (Figure 2A) of XT1 in the aerial parts, the weight of which increased by 20% (*p* ≤ 0.001), particularly in XT1-treated plants as compared to non-inoculated plants (C); both parameters were also significantly higher in XT1-treated *B. cinerea*-infected plants (XT1+Bc) in relation to the *B. cinerea*-infected control and non-treated plants (Bc). No increase in radicular weight was observed following foliar treatment (Figure 2C).

### 3.2. Biocontrol Effect of Strain XT1 on Tomato and Strawberry Plants

**Figure 3:**
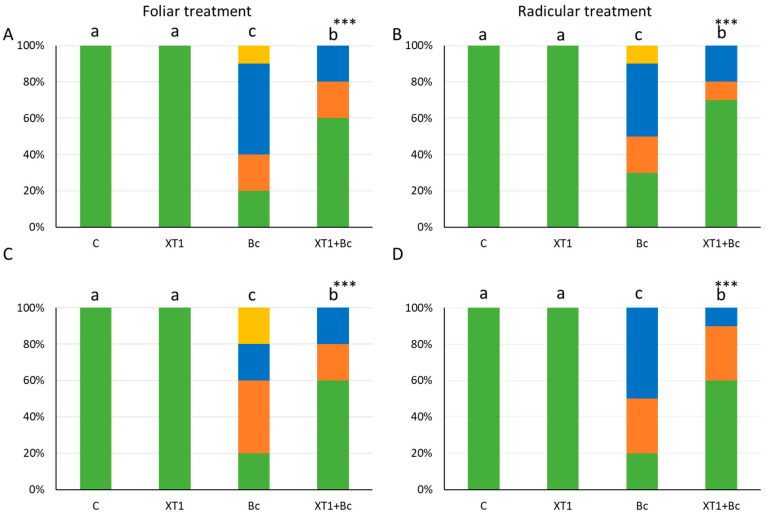
Severity of damage (%) caused by *B. cinerea* in tomato (**A**,**B**) and strawberry (**C**,**D**) plants after foliar and radicular treatment with XT1. C: non-inoculated control plants; XT1: XT1-treated plants; Bc: *B. cinerea*-infected non-treated plants; XT1+Bc: XT1-treated *B. cinerea*-infected plants. Statistical differences according to the Kruskal–Wallis test *** (*p* ≤ 0.001). Significant differences between treatments are indicated by different letters (a,b,c).

The progression of gray mold disease caused by *B. cinerea* in infected tomato and strawberry plants was recorded after inoculation with the pathogen by determining disease incidence (DI) and the severity index for each treatment. In both tomato and strawberry crops, DI in the *B. cinerea*-infected plants (Bc) was over 70%. Moreover, the severity index exceeded 32% in all cases (Figure 3). Foliar and radicular applications of XT1 were found to reduce the severity of fungal pathogen infection. Specifically, both plants showed a sharp reduction of 50% in DI (data not shown) and of 60% in severity in infected XT1-treated *B. cinerea*-infected plants (XT1+Bc) with respect to infected non-treated plants (Bc) (Figure 3).

### 3.3. Effect of XT1 Treatment on Oxidative Status in Tomato and Strawberry Plants

**Figure 4:**
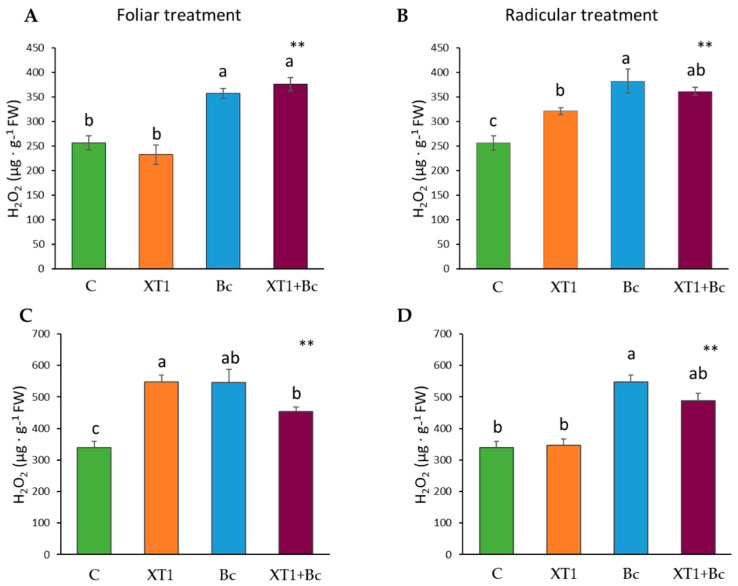
H_2_O_2_ content in tomato (**A**,**B)** and strawberry (**C**,**D**) plants after foliar and radicular XT1 treatment. C: non-inoculated control plants; XT1: XT1-treated plants; Bc: *B. cinerea*-infected non-treated plants; XT1+Bc: XT1-treated *B. cinerea*-infected plants. Letters above the main bars represent statistical differences according to the Fisher test ** (*p* ≤ 0.01). Significant differences between treatments are indicated by different letters (a,b,c).

In this study, membrane damage caused by oxidative stress was estimated by analyzing the superoxide anion H_2_O_2_, as well as malondialdehyde (MDA) levels. The results for hydrogen peroxide in tomato plants revealed significant differences between radicular treatment with XT1-treated and non-inoculated plants (C) (Figure 4B). However, this treatment only produced a slight decrease in XT1-treated *B. cinerea*-infected tomato plants (XT1+Bc) as compared to infected non-treated plants (Bc) (Figure 4B). No differences were found in foliar-treated tomato plants (Figure 4A).

Unlike tomato plants, the foliar treatment of strawberry plants was responsible for differences in H_2_O_2_ (*p* ≤ 0.01) levels between non-inoculated (C) and XT1-treated plants (XT1) (Figure 4C). Likewise, the foliar treatment of strawberry plants reduced H_2_O_2_ levels (*p* ≤ 0.01) in XT1-treated *B. cinerea*-infected plants (Xt1+Bc) as compared to *B. cinerea*-infected non-treated plants (Bc) (Figure 4C), while radicular treatment produced only a slight reduction in hydrogen peroxide between these two types of treatments (Figure 4D).

**Figure 5:**
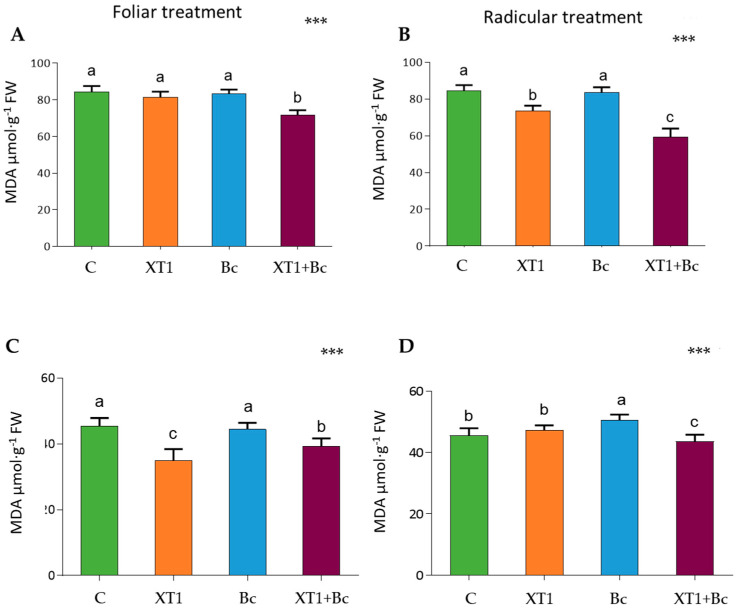
Malondialdehyde (MDA) content in tomato (**A**,**B**) and strawberry (**C**,**D**) plants after foliar and radicular XT1 treatments. C: non-inoculated control plants; XT1: XT1-treated plants; Bc: *B. cinerea*-infected non-treated plants; XT1+Bc: XT1-treated *B. cinerea*-infected plants. Letters above the main bars represent statistical differences according to the Fisher test *** (*p* ≤ 0.001). Significant differences between treatments are indicated by different letters (a,b,c).

Cell damage caused by lipid peroxidation in leaves was estimated by measuring the levels of MDA. The XT1-treated plants (XT1) showed low MDA levels in root-treated tomato (Figure 5B) and foliar-treated strawberry plants (Figure 5C) as compared to non-inoculated plants (C). The results for both plants and both foliar and radicular applications of XT1 indicate a reduction in MDA levels in XT1-treated *B. cinerea*-infected plants (XT1+Bc) with respect to infected non-treated plants (Bc) (*p* ≤ 0.001) (Figure 5).

### 3.4. Callose Deposition in Tomato and Strawberry Plants

**Figure 6:**
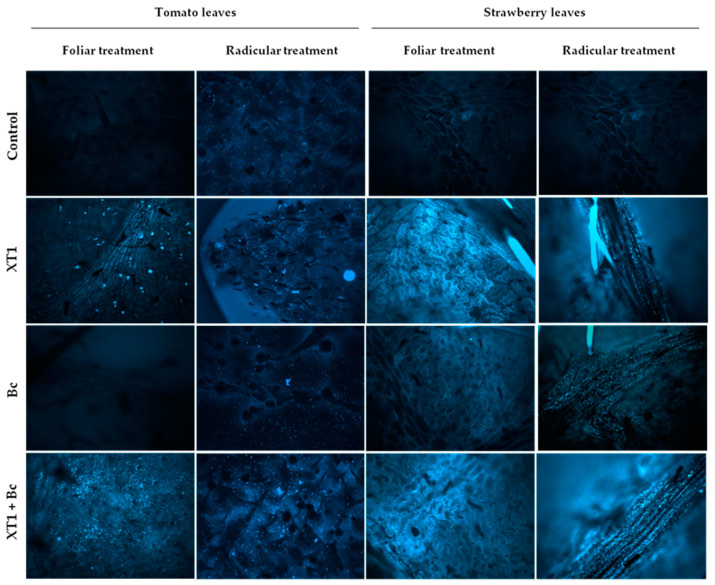
Callose deposition in tomato and strawberry plants after XT1 treatment and *B. cinerea* infection. Control: non-inoculated plants; XT1: XT1-treated plants; Bc: *B. cinerea*-infected non-treated plants; XT1+Bc: XT1-treated *B. cinerea*-infected plants. Optical parameters: magnification 20×; gain 2.1; exhibition 1.06; gamma 3.06; color 471.

Preventive foliar and radicular applications of strain XT1 in *B. cinerea*-infected (XT1+Bc) tomato and strawberry plants increased callose deposits considerably in leaves as compared to infected non-treated plants (Bc). This increase was also pronounced in XT1-treated non-*B. cinerea*-infected plants (XT1) compared to uninoculated plants (C) (Figure 6).

### 3.5. Evaluation of XT1-Induced Resistance against B. cinerea in Tomato and Strawberry Leaves

**Figure 7:**
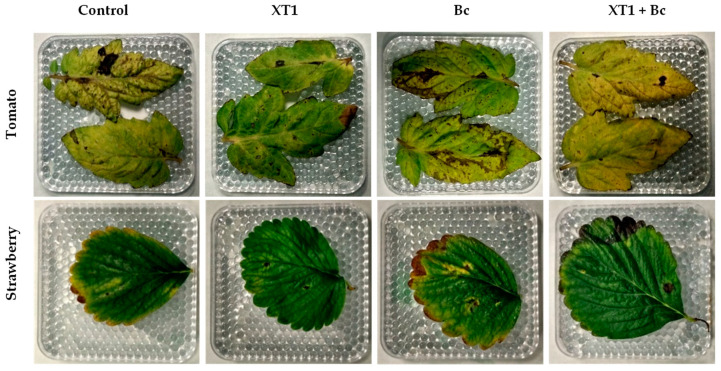
Effect of inoculation with strain XT1 on leaves from *B. cinerea*-infected strawberry and tomato plants. Leaves from negative control (C), XT1-treated (XT1), *B. cinerea*-infected non-treated (Bc) and XT1-treated *B. cinerea*-infected (XT1+Bc) tomato and strawberry plants.

The effectiveness of strain XT1 in increasing tomato and strawberry resistance against *B. cinerea* was tested. Detached leaves of non-inoculated and XT1 root-inoculated plants were infected with the pathogen (Figure 7). Leaves belonging to XT1-treated plants (XT1) and XT1-treated *B. cinerea*-infected plants (XT1+Bc) showed minor symptoms of damage (brown spots and necrosis) of the infected area as compared to those from non-inoculated (C) and *B. cinerea*-infected (Bc) plants. Specifically, the damage caused around the infection zone decreased by 85% in tomato and by 75% in strawberry leaves from the XT1+Bc treatments compared to the Bc treatment.

### 3.6. Effect of Treatment with XT1 on Phytohormone Content

**Figure 8:**
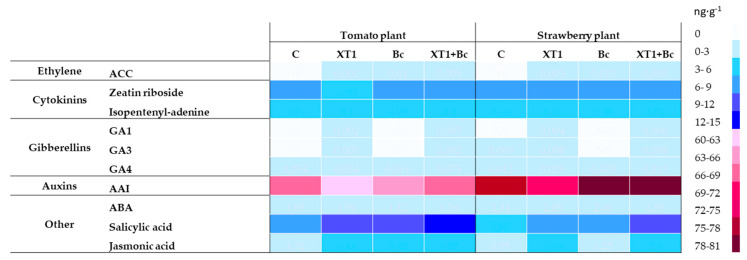
Effect of XT1 inoculation and *B. cinerea* infection on phytohormone content in tomato and strawberry plants. C: non-inoculated control plants; XT1: XT1-treated plants; Bc: *B. cinerea*-infected non-treated plants; XT1+Bc: XT1-treated *B. cinerea*-infected plants.

Radicular applications of XT1, Bc and XT1+Bc increased the levels of the phytohormones ethylene, JA and SA as compared to non-inoculated plants (C) (Figure 8). As shown in the heat map, the application of strain XT1 did not affect the content of hormones gibberellin A4 (GA4), ABA and the cytokinin isopentenyl-adenine. On the other hand, the preventive application of XT1 (XT1) resulted in three-fold higher JA levels as compared to non-inoculated plants (C). Under the conditions described above, the level of SA in tomato and strawberry plants was observed to increase by approximately 20% and 35%, respectively. Although ethylene content rose slightly, a significant increase in SA and JA levels was observed in *B. cinerea*- infected XT1-treated strawberry plants (XT1+Bc) with respect to infected non-treated strawberry plants (Bc). In the case of tomato plants, the JA values were the only ones increased by the preventive use of the strain XT1 (XT1+Bc) compared to infected plants (Bc). On the other hand, radicular applications of XT1 induced the production of the gibberellins GA1 and GA3 in tomato plants, but produced only GA1 in the case of strawberry plants. Finally, according to the results, the treatment with the strain XT1 (XT1) reduced the content of auxins compared with uninoculated tomato and strawberry plants (C).

## 4. Discussion

Although treatments based on extensive pesticide use are usually carried out to control gray mold caused by *B. cinerea*, many restrictions are imposed on these products within the framework of EU pesticide regulation No 1107/2009 [47,48]. In addition, pesticide use involves the production of waste and contaminants from groundwater that increase human health and environmental risks [6,49,50]. Thus, one of the major challenges facing sustainable agriculture is the development of alternatives that respect the environment and safeguard human health. Over the past decade, bacterial strains have been increasingly used as biological control agents given their broad spectrum of action against phytopathogens, as well as their eco-friendly properties.

*B. velezensis* strain XT1 was isolated in 2001 from the rhizosphere of a *Juncus effusus* (soft rush), which was grown in a saline soil (histosol; Soil Survey Staff, 2010) adjacent to the Capacete lagoon (Málaga, Spain) and patented by the University of Granada under Bucharest Convention guidelines, was deposited in the Spanish Type Culture Collection as CECT 8661 and licensed to Xtrem Biotech S.L. [51]. In this study, we evaluated the potential use of XT1 as a plant growth promoter and biocontrol agent against *B. cinerea.* The effect of the fungus on tomatoes and strawberries, two economically important crops, was evaluated [11,52,53,54].

The plant growth-promoting capacity of strains belonging to the genus *Bacillus* has been widely demonstrated [20,55,56,57,58,59], and *B. velezensis* strain BAC03 has been found to enhance the growth of nine different plant types and to increase biomass [58]. On the other hand, *B. velezensis* strain *CBMB205* increases the root length of tomato, red pepper and canola plants by 38.3, 4.2 and 22.4%, respectively [60]. Previous studies have also demonstrated that *B. velezensis* strain XT1 enhances the growth capacity of several crops [61]. However, a study by Palencia et al. (2015) [62] on the effect of treatment with *B. velezensis* strain IT45 on strawberry plants, found that the growth promotion was low in comparison with the uninoculated plants.

Growth promotion capacity has, in some cases, been linked to the mode of application used. Salvatierra-Martínez et al. (2018) [13] demonstrated that only radicular applications of *B. subtilis* strains BBC023 and BBC047 are capable of enhancing biomass production. Similarly, Esitken et al. (2010) [63] found that not all PGPR strains of the genus *Bacillus* act in a similar fashion in strawberry plants and that crop yields, in some cases, increase following root applications of *Bacillus* strain M3 and, in other cases, after foliar applications of *Bacillus* OSU142.

Our results demonstrate that radicular applications of strain XT1 (XT1) induce higher total biomass levels in strawberry plants and a slight increase in tomato plants as compared to uninoculated plants (C). We also show that foliar treatment with XT1 in strawberry plants only significantly affects the aerial biomass. All these results show that growth-promoting activity is closely linked to the mode of application of *B. velezensis.*

With regard to fungal mycelium growth inhibition, several strains of *B. velezensis* have been reported to suppress the in vitro growth of fungal phytopathogens such as the biotrophic/saprotrophic *Trichoderma*, the hemibiotrophic *Fusarium* and the necrotrophics *Alternaria* and *Monilinia* [64,65,66]. In two previous studies, we demonstrated that strain XT1 inhibits the mycelium growth of *Alternaria alternata*, *Fusarium oxysporum*, *Magnaporthe oryzae*, *Sclerotinia sclerotiorum* and *Thanatephorus cucumeris* by over 40%. Antagonistic activity against the pathogens *Monilinia fructicola* [61] and *B. cinerea* (60%) reached maximum levels of > 80% and 60%, respectively, mainly due to the large amount of lipopeptides produced (10 g/L) [67]. Many studies of olive, tomato, corn, peanut, pepper, maize and rice crop yields have also described the biocontrol activity of *B. velezensis* strains against phytopathogens such as *Verticillium dahliae*, *Fusarium graminerarum*, *Sclerotium rolfsii*, *Phytophthora* and *B. cinerea* [68,69,70,71,72,73,74]. Our results are in line with the findings of Lee et al. (2006) [75] who investigated the effect of radicular applications of *B. subtilis* WXCDD105 on *B. cinerea* in tomato plants. However, studies by Salvatierra-Martínez et al. (2018) [13] of radicular applications of *B. subtilis* strains detected low antifungal activity. Little is known about the antifungal impact of root treatments on *B. cinerea* biocontrol activity in strawberry plants. Current research mainly focuses on foliar applications of other *Bacillus* strains, such *B. amyloliquefaciens* FZB42 (formerly *B. velezensis*), *B. amyloliquefaciens*, *B. subtilis*, *B. licheniformis* and *B. megaterium* [53,76,77]. In this study, in vivo experiments with tomato and strawberry crops confirmed the reduction in gray mold incidence and the severity of damage (around 50–60%) caused by *B. cinerea* in tomato and strawberry crops following applications of strain XT1.

Previous studies have highlighted the crucial role played by surfactins and 2–3 butanediol as antimicrobial agents and as compounds which activate induced systemic resistance (ISR) and promote plant growth [32,78,79,80,81]. Strain XT1 produces these compounds [67] and other metabolites [61] which facilitate its antifungal and PGPR activities. The successful antifungal results following radicular applications of strain XT1, as well as its PGP capacity, can be ascribed, not only to the anti-pathogenic action of metabolites, but also to the application method used, which plays an important role in controlling the pathogen *B. cinerea.*

The mechanisms involved in the rapid production of plant cellular defense responses to pathogens include rapid H_2_O_2_ accumulation [82], which is known to be a hallmark event induced by ISR-triggering bacteria [35,83]. In order to restrict pathogenic infection, H_2_O_2_ accumulation is involved in mechanisms such as plant cell wall modifications, signaling stress, systemic acquired resistance (SAR) system and hypersensitive response (HR) activation, defensive gene induction, phytoalexin synthesis and ultimately cell death initiation [84,85,86,87]. We observed an increase in hydrogen peroxide following treatment with XT1 (XT1) in some cases, as well as slight a decrease in this compound in infected and treated plants (XT1+Bc). Previous studies have reported that the early activation of defense systems, and thus H_2_O_2_ accumulation, boosts resistance against *B. cinerea* in tomato and strawberry plants [84,87,88,89]. Although hydrogen peroxide accumulation has been widely shown to contribute to defense against pathogens, different studies have shown that virulence factors in necrotrophic organisms such as *B. cinerea* play a very important role in the induction of unregulated reactive oxygen species (ROS) production [86,87], which explains the high levels of ROS in *B. cinerea*-infected plants observed in this study. Nie et al. (2017) [90] found that after infection with *B. cinerea*, plants pretreated with *B. cereus* AR156 accumulated higher H_2_O_2_ levels than infected non-treated plants, which enhanced and accelerated the defense response. However, we observed different results. Excessive ROS accumulation can be harmful to plants, leading to oxidative stress damage to their deoxyribonucleic (DNA) acids, proteins, chlorophylls and membrane functions. Thus, H_2_O_2_ levels [91] need to be optimally balanced to prevent excessive ROS accumulation in *B. cinerea*-infected XT1-treated plants.

Apart from hydrogen peroxide, malondialdehyde (MDA) is another parameter associated with plant oxidation status. MDA determines the lipid peroxidation produced by ROS, which directly affects the cellular lipid membrane, leading to oxidative degradation and cell lysis [92,93]. Preventive foliar and root applications of *B. velezensis* strain XT1 reduce the MDA content in *B. cinerea*-infected tomato and strawberry plants (XT1+Bc). Although similar results have been obtained using different *Bacillus* strains and crops [92,94], to our knowledge, no previous studies demonstrate the ability of *B. velezensis* to reduce MDA levels in plants affected by *B. cinerea,* a mechanism which might explain the in vivo antifungal activity of strain XT1.

As with hydrogen peroxide, callose accumulation is regarded as a defense response induced by ISR-triggering bacteria [35,83]. Preventive foliar and radicular applications of strain XT1 in *B. cinerea*-infected tomato and strawberry plants (XT1+Bc) resulted in a considerable increase in callose deposits. Cell wall reinforcement caused by increased callose deposition has been widely demonstrated to be an important defense against the development of *B. cinerea* [86,88,90]. However, little is known about the induction of callose deposition by other *Bacillus* species in response to *B. cinerea* infection in tomato and strawberry plants. In order to test the effectiveness of *B. velezensis* XT1 in increasing tomato resistance to *B. cinerea* in tomato and strawberry plants, infection bioassays were carried out on leaves. Remarkably, inoculation with XT1 resulted in a significant reduction in disease symptoms in both crops.

The phytohormones salicylic acid (SA), jasmonic acid (JA), abscisic acid (ABA) and ethylene (ET) play a fundamental role in the initiation and regulation of the plant defense mechanisms mentioned above [38,39,40]. More specifically, plant defense responses to *B. cinerea* are coordinated by the interplay between JA- and SA-regulated signaling pathways [8], the two major branches of defense-related signaling. On the whole, the coordination of defenses against biotrophic pathogens is mediated by SA, while the JA/ET tandem is associated with protection against necrotrophic pathogens, including *Botrytis* [41].

Our results, which are in line with previous studies, show that infection with *B. cinerea* in tomato and strawberry plants triggers the production of ET, JA and SA. Specifically, the increase in SA could be attributed to the activation of plant systemic acquired resistance (SAR) in response to *B. cinerea* infection, which promotes mechanisms that regulate defense systems against pathogens [90].

In recent years, there has been increasing interest in the development and use of bacteria capable of promoting plant ISR against various pathogens. Niu et al. (2011) [85] have documented the ability of *B. cereus* strain AR156 to activate ISR in *Arabidopsis thaliana* and consequently to accelerate the activation of cellular defense responses following infection with *P. syringae* pv tomato DC3000. Very similar results were obtained by Nie et al. (2017) [90] and Ahn et al. (2007) [83], who highlighted the fundamental role of the JA/ET-mediated signaling pathway in combating *B. cinerea* and *P. syringae* pv tomato. Elicitors synthesized by PGPR microorganisms capable of activating ISR include the lipopeptides surfactin, bacillomycin and phengicine, as well as the volatile organic compound 2,3-butanediol [21,28,35,79]. Foliar and radicular applications of strain XT1 on infected strawberry and tomato plants (XT1+Bc) modified phytohormone content considerably, with a high increase in JA levels. Given the ability of the biocontrol agent to reduce the infection caused by the phytopathogen and given the activation of the defense mechanisms detailed above, the increase in plant hormonal levels in non-infected and XT1-treated plants could be due to *priming*. Although our findings, with respect to phytohormone content in non-infected and infected XT1-treated plants, suggest that the JA/ET system is involved in the improvement of these defense systems, this fact needs to be confirmed through a more detailed analysis of signaling mechanisms triggered by the biological control agent XT1.

## 5. Conclusions

Greenhouse experiments on tomato and strawberry plants confirm that preventive applications of strain XT1 are capable of reducing the incidence and severity of *B. cinerea*-induced damage by over 50%. In addition, our results show that treatment with strain XT1 improves plant growth and development in both infected and non-infected plants. The evaluation of the foliar and radicular modes of application suggests that the type of application used plays a fundamental role in the effectiveness of the biocontrol agent. According to our findings, while foliar applications of strain XT1 only led to high levels of antifungal activity, root applications of this biocontrol agent increased both plant biomass and protection against *B. cinerea*. Finally, the analysis of stress parameters and phytohormone content indicate that preventive applications of strain XT1 in plants following infection with *B. cinerea* promote the activation of defense mechanisms in strawberry and tomato plants. In addition, given the hormone levels observed, the signaling and activation pathways of these defense mechanisms could be mediated by the JA/ET pathway. Therefore, we could assume that the strain XT1 plays an important role in protecting against *B. cinerea* through the activation of induced systemic resistance (ISR) with the accumulation of callose deposits on cell walls and H_2_O_2_ production.
